# Increment of body mass index is positively correlated with worsening of endothelium-dependent and independent changes in forearm blood flow

**DOI:** 10.3389/fphys.2015.00223

**Published:** 2015-08-11

**Authors:** Luiz G. Kraemer-Aguiar, Marcos L. de Miranda, Daniel A. Bottino, Ronald de A. Lima, Maria das Graças C. de Souza, Michelle de Moura Balarini, Nivaldo R. Villela, Eliete Bouskela

**Affiliations:** ^1^Endocrinology, Obesity Unit, Policlínica Piquet Carneiro, Department of Internal Medicine, Faculty of Medical Sciences, Rio de Janeiro State UniversityRio de Janeiro, Brazil; ^2^Laboratory for Clinical and Experimental Research in Vascular Biology - BioVasc, Biomedical Center, Rio de Janeiro State UniversityRio de Janeiro, Brazil; ^3^Critical Care, Department of Internal Medicine, Faculty of Medical Sciences, Rio de Janeiro State UniversityRio de Janeiro, Brazil; ^4^Department of Anesthesiology, National Cancer Institute HospitalRio de Janeiro, Brazil; ^5^Department of Internal Medicine, Andarai Federal HospitalRio de Janeiro, Brazil; ^6^Anesthesiology, Department of Surgery, Faculty of Medical Sciences, Rio de Janeiro State UniversityRio de Janeiro, Brazil

**Keywords:** obesity, overweight, endothelial function, vascular reactivity, venous occlusion plethysmography, forearm blood flow

## Abstract

Obesity is associated with the impairment of endothelial function leading to the initiation of the atherosclerotic process. As obesity is a multiple grade disease, we have hypothesized that an increasing impairment of endothelial and vascular smooth muscle cell functions occurs from lean subjects to severe obese ones, creating a window of opportunities for preventive measures. Thus, the present study was carried out to investigate the grade of obesity in which endothelial dysfunction can be detected and if there is an increasing impairment of endothelial and vascular smooth muscle cell functions as body mass index increases. According to body mass index, subjects were allocated into five groups: Lean controls (*n* = 9); Overweight (*n* = 11); Obese class I (*n* = 26); Obese class II (*n* = 15); Obese class III (*n* = 19). Endothelial and vascular smooth muscle cell functions were evaluated measuring forearm blood flow responses to increasing intra-arterial infusions of acetylcholine and sodium nitroprusside using venous occlusion plethysmography. We observed that forearm blood flow was progressively impaired from lean controls to severe obese and found no significant differences between Lean controls and Overweight groups. Known determinants of endothelial dysfunction, such as inflammatory response, insulin resistance, and diagnosis of metabolic syndrome, did not correlate with forearm blood flow response to vasodilators. Moreover, several risk factors for atherosclerosis were excluded as independent predictors after confounder-adjusted analysis. Our data suggests that obesity *per se* could be sufficient to promote impairment of vascular reactivity, that obesity class I is the first grade of obesity in which endothelial dysfunction can be detected, and that body mass index positively correlates with the worsening of endothelium-dependent and independent changes in forearm blood flow.

## Introduction

Obesity is the worldwide leading metabolic disease with progressively increasing prevalence in developed and developing countries. Due to the rising trend in its prevalence, by the year 2030, if no actions are taken against this threat, the number of obese adults is projected to be around 600 million to 1 billion individuals (Kelly et al., 2008). This alarming situation made the World Health Organization refers to obesity as a global epidemic (Formiguera and Cantón, 2004).

Besides being an important clinical and public health burden in itself, obesity represents an important risk factor for atherosclerosis-related cardiovascular diseases, such as coronary artery disease. Indeed, some studies have indicated that obesity is associated with the impairment of endothelial function, one of the earliest markers of the atherosclerotic process (Hashimoto et al., 1998; de Jongh et al., 2004; Anderson, 2007; Yeboah et al., 2007). In this pathophysiological model of atherosclerotic disease, before the development of clinically overt atherosclerosis, arterial wall changes are limited to impaired endothelial function (Hashimoto et al., 2000).

As obesity is a multiple grade disease, we have hypothesized that an increasing impairment of endothelial and vascular smooth muscle cell functions occurs from lean subjects to severe obese ones, creating a window of opportunities for preventive measures. In fact, previous studies support the idea of the existence of a reversible early stage of atherosclerosis (Hashimoto et al., 2000). Unfortunately, although our current knowledge offers a potential explanation for the relationship between obesity and atherosclerosis, it still lacks the grade of obesity in which endothelial dysfunction begins. Thus, the present study was carried out to investigate the grade of obesity in which endothelial dysfunction can be detected and if there is an increasing impairment of endothelial and vascular smooth muscle cell functions as body mass index increases. Additionally, in order to perform a confounder-adjusted analysis we have tried to correlate our findings with known determinants of endothelial dysfunction, such as markers of inflammatory response, insulin resistance, and diagnosis of metabolic syndrome.

## Materials and methods

### Subjects

Eighty women, aged 18–30 years old, with sedentary lifestyle were enrolled in this study. These subjects were consecutively recruited from Rio de Janeiro State University's outpatient clinic and from community volunteers. A structured interview, complete physical examination, and laboratory tests were performed to exclude subjects with diseases other than obesity, hypertension, hyperlipidemia, insulin resistance, and metabolic syndrome. Patients with glucose intolerance, diabetes mellitus, smoking habit, regular drinking habit, regular use of medications, and clinical manifestations of atherosclerosis were intentionally excluded due to known changes of vascular function.

The Ethics Committee of Pedro Ernesto University Hospital (Rio de Janeiro State University, Rio de Janeiro, Brazil) approved the study design and protocol and, after receiving a thorough explanation of the study, each subject gave written informed consent before enrollment.

### Study design

Each subject made two visits (on two consecutive days) to the Laboratory for Clinical and Experimental Research in Vascular Biology at Rio de Janeiro State University. In the morning of the first clinical visit, they were subjected to physical examination, anthropometric measurements, and laboratory tests. Blood and urine sampling was performed after 10–12 h overnight fast to measure plasma glucose, lipid profile, insulin, and other biochemical and inflammatory parameters. After fasting blood sampling, all subjects underwent a 2-h 75-g oral anhydrous glucose tolerance test to exclude glucose intolerance or diabetes (according to American Diabetes Association criteria, American Diabetes Association, 2014). In the morning of the second clinical visit, venous occlusion plethysmography was performed after 8–12 h overnight fast to assess endothelial function and vascular reactivity.

### Physical examination, anthropometric measurements, and laboratory tests

Arterial blood pressure was measured twice in supine position with 5-min interval between measurements, using an automated apparatus (LifeWindow LW6000, Digicare Biomedical Technology, West Palm Beach, FL, USA). A trained examiner collected all anthropometric measurements in duplicate: waist was measured at its smallest point with a relaxed abdomen, hip at the widest part of gluteal region, height using a vertical bar stadiometer, and weight using a digital scale (Filizola, São Paulo, SP, Brazil). Body mass index (BMI) was defined as the ratio between weight in Kg and squared height in meters.

All laboratory measurements were performed in duplicate. Plasma glucose level was assayed by the glucose oxidase method and serum insulin level by electrochemiluminescence. Serum non-esterified fatty acids (NEFA), serum total cholesterol, and serum triglyceride levels were measured enzymatically. Serum high-density lipoprotein (HDL) cholesterol level was measured by the heparin-Ca^2+^/Ni^2+^ precipitation method. Serum C-reactive protein was measured by immunoturbidimetry. Urinary 8-isoprostane, serum interleukin-6 (IL-6), serum leptin, serum adiponectin, and serum resistin were assayed by ELISA. 8-isoprostane was measured in urine due to methodological problems with its serum measurement.

Low-density lipoprotein (LDL) cholesterol level was calculated according to Friedwald equation. In order to evaluate the insulin resistance status, the Homeostasis Model Assessment (HOMA-IR) was calculated according to the equation: HOMA-IR = [Plasma Glucose (in mmol/L) × Insulin (in μUI/mL)/22.5], using fasting levels.

### Metabolic syndrome diagnosis

Metabolic syndrome was diagnosed according to NCEP-ATPIII criteria (National Cholesterol Education Program (NCEP) Expert Panel on Detection, Evaluation, and Treatment of High Blood Cholesterol in Adults (Adult Treatment Panel III), 2002), which defines metabolic syndrome when three or more of the following criteria are present: 1, waist circumference ≥88 cm; 2, triglycerides ≥150 mg/dL; 3, HDL < 50 mg/dL; 4, fasting plasma glucose concentration ≥100 mg/dL; 5, arterial blood pressure ≥130/85 mmHg.

### Venous occlusion plethysmography

Studies were carried out in a quiet temperature-controlled room (21 ± 1°C) with the subjects in supine position. Forearm blood flow (FBF), in mL.100 mL^−1^ forearm volume.min^−1^, was measured by mercury-in-silastic strain gauge venous occlusion plethysmograph (Hokanson EC6, D.E. Hokanson, Bellevue, WA, USA) placed in the point of maximal circumference of the non-dominant forearm, which was maintained at the level of the heart. Drugs or normal saline were continuously infused at 1.0 mL.min^−1^ into the ipsilateral brachial artery through a 27 SWG needle introduced under local anesthesia (1 mL of 1% lidocaine). After arterial needle insertion, subjects were allowed to rest for 30 min before any flow measurement.

Endothelial and vascular smooth muscle cell functions, as depicted by vascular reactivity, were evaluated measuring FBF responses to intra-arterial infusion of three increasing doses of endothelium-dependent [acetylcholine (Ach) 7.5, 15, and 30 μg.min^−1^] and independent [sodium nitroprusside (SNP) 2, 4, 8 μg.min^−1^] vasodilators, in this order. Each dose was infused for 5 min and FBF was recorded during the last 2 min of infusion. Prior to initiation of Ach and SNP, normal saline was infused for 20 min, and then blood flow measurements were taken to establish resting control values (baseline). This allowed an interval of 20 min between Ach and SNP infusions.

During the recording periods, hand circulation was excluded by inflating a wrist cuff to suprasystolic pressure 1 min before flow measurement. The upper arm congesting cuffs were intermittently inflated to 40 mmHg for 10 s in 15 s cycles to temporally occlude venous return, producing five flow measurements (slopes). Venous occlusion plethysmography apparatus was connected to an analog-to-digital converter (PowerLab 8/35, AD Instruments, Castle Hill, Australia) and data were recorded directly onto computer for later analysis with LabChart Pro 8 software (AD Instruments, Castle Hill, Australia). The first flow measurement was always excluded and the mean of the remaining four measurements in each recording period was used for data analysis. Responses to each dose of Ach or SNP were summed and analyzed as Cumulative FBF, which represents an integrated measurement of the cumulative response to the progressive doses of each vasodilator.

During FBF measurement, arterial blood pressure was measured in contralateral arm using a semiautomated oscillometric device (LifeWindow LW6000, Digicare Biomedical Technology, West Palm Beach, FL, USA).

### Statistical analysis

Results are expressed as means ± standard deviation of the mean (SD) for each group, unless otherwise noted. Statistical comparisons of normally distributed variables were performed using One-Way ANOVA, whereas Kruskal-Wallis test was used for other variables. When appropriate, an adequate test was used for *post-hoc* analysis: Bonferroni method or Dunn's multiple-comparisons. We also have performed univariate and multiple regression analysis of cumulative FBF responses to intra-arterial infusion of vasodilators in relation to other study variables or set of variables in order to find significant correlations and to perform confounder-adjusted analysis. Spearman correlation coefficients and *p*-values were calculated for univariate analysis. All statistical analyses were performed using Stata 10.1 (StataCorp, College Station, TX, USA) and GraphPad Prism 6.03 (GraphPad Software, La Jolla, CA, USA) and the significance level was set as *p* < 0.05 for a two-tailed test.

## Results

According to BMI, subjects were allocated into five groups for data analysis: 1, Lean controls (*n* = 9; BMI = 22.2 ± 1.3 kg.m^−2^); 2, Overweight (*n* = 11; BMI = 27.5 ± 1.4 kg.m^−2^); 3, Obese class I (*n* = 26; BMI = 32.3 ± 1.4 kg.m^−2^); 4, Obese class II (*n* = 15; BMI = 37.6 ± 1.6 kg.m^−2^); 5, Obese class III (*n* = 19; BMI = 44.0 ± 2.4 kg.m^−2^). Clinical, anthropometric, and laboratory characteristics of groups are presented in Table 1.

**Table 1:** **Clinical, anthropometric, and laboratory characteristics of groups**.

	**Lean controls**	**Overweight**	**Obese class I**	**Obese class II**	**Obese class III**
Age (years)	22.7 ± 3.3	26.2 ± 3.2	25.0 ± 3.2	25.5 ± 3.4	25.9 ± 3.1
Weight (kg)	55.4 ± 5.5^#^	73.5 ± 5.3^#^	83.0 ± 9.1^#^	96.0 ± 10.0^#^	113.9 ± 13.2^#^
Waist (cm)	66.8 ± 4.6^#^	85.9 ± 6.6^#^	95.2 ± 8.1^#^	102.1 ± 8.3^#^	111.9 ± 9.5^#^
Hip (cm)	94.7 ± 6.9	104.6 ± 6.9	112.5 ± 6.0^*^	122.0 ± 7.6^*^	132.6 ± 10.5^*^
Systolic blood pressure (mmHg)	108.9 ± 9.0	118.8 ± 13.7	128.3 ± 10.8^*^	129.1 ± 12.4^*^	133.0 ± 15.8^*^
Diastolic blood pressure (mmHg)	68.7 ± 10.1	76.2 ± 10.9	76.1 ± 8.6	76.3 ± 6.5	77.1 ± 10.2
Fasting plasma glucose (mmol.L^−1^)	4.6 ± 0.2	4.8 ± 0.2	4.9 ± 0.2	4.8 ± 0.2	4.9 ± 0.1
Serum insulin (μUI.mL^−1^)	5.7 ± 2.7	11.5 ± 5.6	15.5 ± 8.3^*^	20.3 ± 10.5^*^	22.3 ± 10.4^*^
HOMA-IR	1.2 ± 0.5	2.6 ± 1.2	3.4 ± 2.0^*^	4.5 ± 2.5^*^	4.8 ± 2.3^*^
Serum total cholesterol (mg.dL^−1^)	180.9 ± 22.9	174.3 ± 34.6	196.5 ± 27.4	168.3 ± 22.6	184.1 ± 35.0
LDL-cholesterol (mg.dL^−1^)	95.2 ± 23.6	99.7 ± 29.1	116.3 ± 26.4	97.3 ± 17.7	110.8 ± 30.5
Serum HDL-cholesterol (mg.dL^−1^)	67.9 ± 12.9	52.2 ± 13.1	53.3 ± 14.4	48.7 ± 10.5^*^	46.7 ± 13.3^*^
Serum triglycerides (mg.dL^−1^)	74.0 ± 25.0	92.5 ± 30.2	134.3 ± 72.6	111.5 ± 52.1	132.6 ± 67.5
Serum leptin (pg.mL^−1^)	11,163 ± 5217	23,086 ± 6190	35,266 ± 20,105^*^	53,534 ± 17,602^*^	49,847 ± 23,798^*^
Serum adiponectin (ng.mL^−1^)	13,205 ± 2660	7965 ± 6996	5789 ± 2154^*^	4401 ± 1848^*^	5275 ± 2339^*^
Serum resistin (ng.mL^−1^)	8.5 ± 3.1	7.5 ± 2.6	7.0 ± 2.9	7.9 ± 3.8	8.4 ± 5.8
Serum NEFA (mmol.L^−1^)	0.4 ± 0.2	0.7 ± 0.1	0.7 ± 0.3	0.8 ± 0.2^*^	0.7 ± 0.2
Serum C-reactive protein (mg.dL^−1^)	0.4 ± 0.3	0.4 ± 0.3	0.9 ± 0.7	1.0 ± 0.7	0.7 ± 0.5
Serum IL-6 (pg.mL^−1^)	1.4 ± 0.2	2.0 ± 0.9	1.9 ± 0.8	3.6 ± 2.4^*^	2.9 ± 1.2^*^
Urinary 8-isoprostane (pg/μmol creatinine)	77.6 ± 18.7	106.6 ± 65.6	114.7 ± 103.4	131.7 ± 107.8	148.0 ± 145.6
Percentage of subjects with metabolic syndrome diagnosis (%)	0%	0%	42%	27%	63%^*^

Results are expressed as means ± SD for each group.

* p < 0.05 as compared with Lean controls group.

# p < 0.05 as compared with any other group.

When compared with lean subjects, obese class I, II, and III ones showed greater systolic blood pressure, serum insulin levels, HOMA-IR, and leptin levels but lower HDL-cholesterol and adiponectin levels. IL-6 was significantly greater in Obese class II and III groups. Obese class III group had a significantly greater percentage of subjects with metabolic syndrome.

### Venous occlusion plethysmography

**Figure 1:**
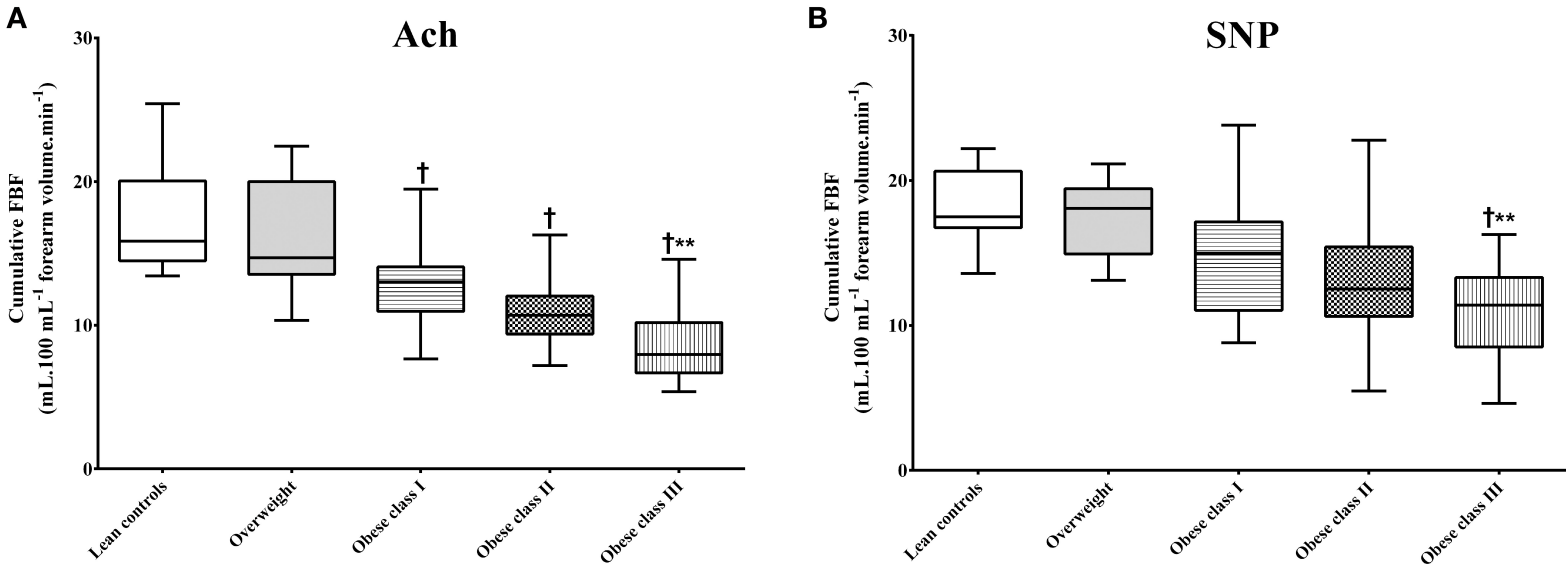
**Cumulative forearm blood flow (FBF) after intra-arterial infusions of Ach or SNP**. **(A,B)** Show the cumulative FBFs derived from the sum of responses to each dose of Ach or SNP. ^†^*p* < 0.05 as compared with Lean controls or Overweight groups. ^**^p < 0.05 as compared with Obese class I group.

Baseline FBF did not significantly differ between groups (Table 2). Ach and SNP intra-arterial infusions were associated with FBF improvements in all groups, but when compared with lean subjects, significant impairment of vascular reactivity was observed in obese class I, II, and III ones (Table 2). A lesser cumulative FBF response to intra-arterial infusion of Ach was observed in obese class I, II, and III subjects when compared with lean and overweight ones (Figure 1A). However, no significant differences were found between lean, overweight, obese class I, and obese class II subjects when cumulative FBF response to intra-arterial infusion of SNP was analyzed (Figure 1B). Comparisons between the three obese groups showed greater impairment of vascular reactivity in obese class III subjects (Table 2 and Figure 1). No significant differences were found between Lean controls and Overweight groups.

**Table 2:** **Forearm blood flow (FBF) at baseline and after all three doses of Ach or SNP**.

	**Lean controls**	**Overweight**	**Obese class I**	**Obese class II**	**Obese class III**
FBF prior to Ach infusion	1.5±0.4	1.7±0.4	2.1±0.6	1.9±0.5	1.7±0.5
FBF after Ach infusion	10.9±2.9	9.4±3.0	6.6±1.5^*^	5.9±1.5^*^	3.7±1.1^#^
FBF prior to SNP infusion	1.6±0.6	1.9±0.2	2.1±0.7	1.8±0.7	2.0±0.7
FBF after SNP infusion	9.6±1.8	9.3±1.6	7.6±2.2^*^	6.7±1.9^*^	5.1±1.5^#^

Results are expressed as means ± SD for each group. FBF is expressed in mL.100 mL^−1^ forearm volume.min^−1^.

* p < 0.05 as compared with Lean controls group.

# p < 0.05 as compared with any other group.

**Figure 2:**
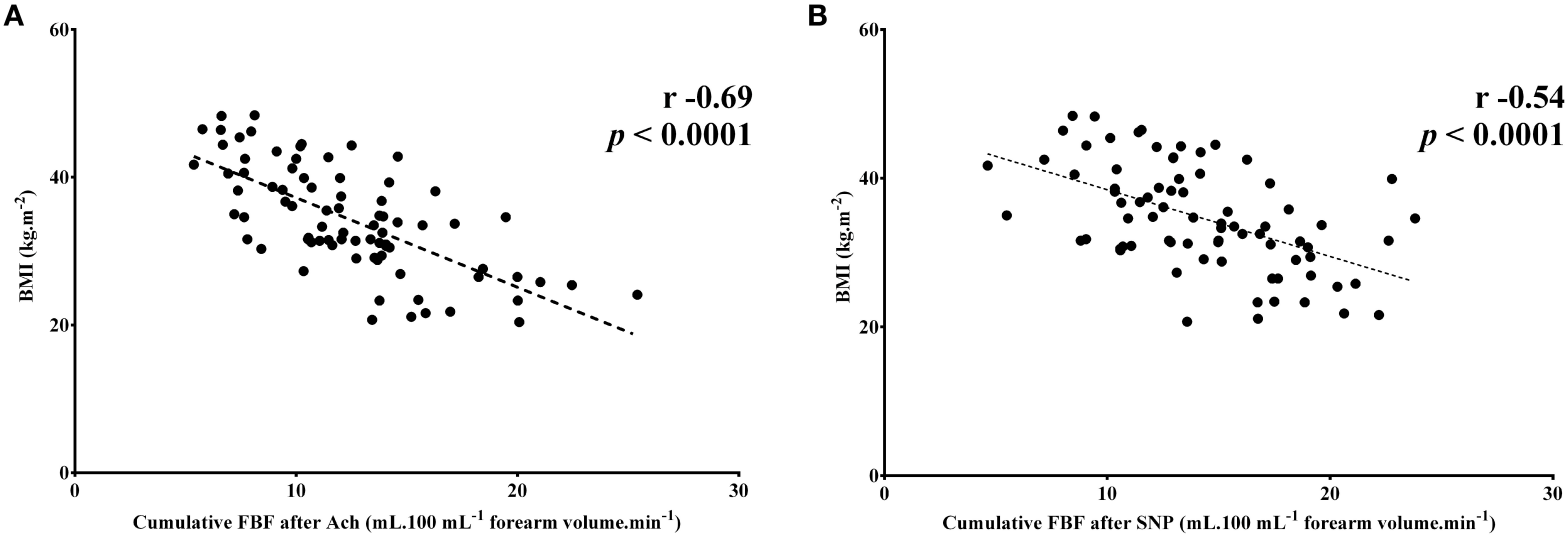
**Scatter plots of significant correlations between cumulative forearm blood flow (FBF) responses to intra-arterial infusion of vasodilators and body mass index (BMI). (A,B)** Note that the less the BMI is, the better the cumulative response to the progressive doses of vasodilators are. Spearman correlation coefficient and p-values for univariate analysis are shown for each correlation. *n* = 80.

On univariate analysis BMI (*r* = −0.69; *p* < 0.0001), systolic blood pressure (*r* = −0.25; *p* = 0.03), waist circumference (*r* = −0.58; *p* < 0.0001), hip circumference (*r* = −0.64; *p* < 0.0001), leptin levels (*r* = −0.54; *p* < 0.0001), and adiponectin levels (*r* = 0.47; *p* = 0.0001) showed significant correlation with the cumulative FBF responses to intra-arterial infusion of Ach, whereas BMI (*r* = −0.54; *p* < 0.0001), systolic blood pressure (*r* = −0.35; *p* = 0.002), waist circumference (*r* = −0.41; *p* = 0.0002), hip circumference (*r* = −0.51; *p* < 0.0001), and leptin levels (*r* = −0.53; *p* < 0.0001) showed significant correlation with the cumulative FBF responses to intra-arterial infusion of SNP. After confounder-adjustment, on multiple regression analysis only BMI showed significant correlation with the cumulative FBF responses to intra-arterial infusion of vasodilators (*R*^2^ = 0.47 for Ach and 0.28 for SNP). Spearman correlation coefficients for univariate analysis of BMI data are presented in Figure 2.

Arterial blood pressure level evolution during vasodilators infusions showed no systemic effects of drugs nor significant differences between groups (data not shown).

## Discussion

This study showed that vascular reactivity to intra-arterial infusions of endothelium-dependent (Ach) and independent (SNP) vasodilators, was greater in lean subjects than in obese ones. Several studies have already indicated that obesity is associated with the impairment of endothelial function (Hashimoto et al., 1998; de Jongh et al., 2004; Anderson, 2007; Yeboah et al., 2007), and that an inflammatory state associated with the release of a variety of cytokines and cytokine-like substances, such as leptin and resistin, is among the mechanisms that underlie the resultant microvascular dysfunction (Singer and Granger, 2007). An altered sympathetic neuronal responsiveness leading to diminished nitric-oxide dilation has also been suggested (Vollenweider et al., 1994). Our new finding was that an increasing impairment of endothelium-dependent and independent changes in FBF could be observed as BMI increases. Statistically, not only univariate analysis but also multiple regression analysis confirmed that BMI is an independent variable relating to cumulative FBF response to vasodilators.

Other important finding of our study was that no statistical differences were observed in vascular smooth muscle cell function between lean, overweight, obese class I, and obese class II subjects (considering cumulative SNP response data) and in both endothelial and smooth muscle cell function between lean subjects and overweight ones. Together, these findings support the hypotheses that obesity class I is the first grade of obesity in which endothelial dysfunction can be detected, that arterial wall damages progress in direct relationship with BMI, and that in initial stages of obesity (obesity class I and II) before the development of overt atherosclerosis with vascular smooth muscle cell function impairment, arterial wall changes are limited to impaired endothelial function. Of note, it has already been shown that implementation of strategies targeting the reduction of risk factors related to atherosclerotic disease resulted in improvements of impaired endothelial function (Celermajer et al., 1993; Vogel, 1999; de Kleijn et al., 2001; Gokce et al., 2002; Taddei et al., 2002), allowing us to hypothesize about the existence of a “potentially reversible stage of atherosclerosis” when preventive measures could be taken. Unfortunately, despite considerable amount of evidence that impaired endothelial function can be improved by risk reduction strategies, no consensus exists that the reversal of endothelial dysfunction is associated with a reduction in cardiovascular events (Taddei et al., 2002).

Interestingly, known determinants of endothelial dysfunction, such as inflammatory response, insulin resistance (using HOMA-IR as a surrogate marker), and diagnosis of metabolic syndrome, did not correlate with FBF response to vasodilators in our study. Moreover, several risk factors for atherosclerosis were excluded as independent predictors after confounder-adjusted analysis. Our results indicated that obesity per se could be sufficient to promote impairment of endothelial function and vascular wall reactivity. The selection of young subjects and exclusion of those with diabetes mellitus, smoking habit, regular use of medications, and clinical manifestations of atherosclerosis, imposing age and disease restrictions to our study design may have helped in dealing with confounding variables, clarifying the role of obesity.

Finally, we have chosen to measure FBF with venous occlusion plethysmography method given its minimally invasive characteristic and our previous experience with this methodology (de Aguiar et al., 2006). Furthermore, systemic administration of vasoactive drugs may lead to central effects, hormonal responses, changes in sympathetic output, and alterations in blood pressure that make changes in forearm blood flow difficult to interpret (Benjamin et al., 1995). Thus, another advantage of our in vivo study model is the possibility to elevate flow locally by intra-arterial infusions of sub-systemic doses of vasodilators (often 100–1000 times lower than a systemically effective dose), obviating any confounding systemic effects on drug response. Drugs are infused through a fine needle placed in the brachial artery allowing the study of the direct vascular effects, without affecting systemic parameters. There is extensive worldwide experience with this technique and it is considered quite safe (Benjamin et al., 1995).

In conclusion, the present study demonstrated that obesity class I is the first grade of obesity in which endothelial dysfunction can be detected, that BMI is an independent variable relating to FBF response to intra-arterial infusions of vasodilators, existing an increasing impairment of such response when BMI increases, and that the worsening of vascular response to vasodilators is limited to an impaired endothelium-dependent response in initial stages of obesity.

### Limitations and perspectives

Our study has some limitations. First, fitting BMI as a predictor in our linear model resulted in low R-squared coefficients, allowing us to hypothesize that other non-measured/studied predictors are present. Those predictors could help to explain part of the variations in vascular reactivity to intra-arterial infusions of vasodilators fitting better linear models. Second, the observational design of our study does not allow further inferences about the reversal of endothelial dysfunction with implementation of preventive measures. Third, although our inclusion/exclusion criteria may have helped in dealing with confounding, it may have decreased the external validity of our findings. Finally, we cannot exclude that the relatively small and unbalanced number of subjects per group may have biased our findings. Further studies are needed to elucidate these issues.

## Author contributions

LK designed the study, recruited patients, performed the experiments, collected and analyzed the data, and critically revised the final version of the manuscript; MM analyzed the data and wrote the final version of the manuscript; DB designed the study, performed the experiments, and collected the data; RL performed the experiments and collected the data; MS performed the experiments and collected the data; MB analyzed the data and wrote the final version of the manuscript; NV designed the study, performed the experiments, and collected and analyzed the data; EB designed the study and critically revised the final version of the manuscript. All authors read and approved the final version of the manuscript.

### Conflict of interest statement

The authors declare that the research was conducted in the absence of any commercial or financial relationships that could be construed as a potential conflict of interest.

## Abbreviations

Ach: acetylcholine
BMI: body mass index
FBF: forearm blood flow
HDL: high-density lipoprotein
HOMA-IR: Homeostasis Model Assessment-Insulin resistance
IL-6: interleukin-6
LDL: low-density lipoprotein
NEFA: non-esterified fatty acids
SNP: sodium nitroprusside.
